# The Unique Genome of the Virus and Alternative Strategies for its Realization

**DOI:** 10.32607/actanaturae.11904

**Published:** 2023

**Authors:** O. P. Zhirnov

**Affiliations:** The N.F.Gamaleya Research Center of epidemiology and microbiology, The D.I. Ivanovsky Institute of Virology, Moscow, 123098 Russian Federation; The Russian-German Academy of medico-social and biotechnological sciences; The Innovation Center of Skolkovo, Moscow, 121205 Russian Federation

**Keywords:** virus diversity, genome strategy, ambisense genes, virus classification

## Abstract

Dedicated to the 130th anniversary of Dmitry Ivanovsky’s discovery of the virus kingdom as a new form of biological life.

The genome of some RNA-containing viruses comprises ambipolar genes that are arranged in stacks (one above the other) encoding proteins in opposite directions. Ambipolar genes provide a new approach for developing viral diversity when virions possessing an identical genome may differ in its expression scheme (strategy) and have distinct types of progeny virions varying in the genomic RNA polarity and the composition of proteins expressed by positive- or negative-sense genes, the so-called ambipolar virions. So far, this pathway of viral genome expression remains hypothetical and hidden from us, like the dark side of the Moon, and deserves a detailed study.

**130** years ago, the outstanding Russian scientist D.I. Ivanovsky reported having discovered a new form of biological life, the so-called *“contagium vivum fixum” *[1, 2], which was later classified into a separate kingdom of viruses [3, 4]. According to the current International Committee on the Taxonomy of Viruses (ICTV) Release (https://ictv.global/taxonomy), the virus domain comprises six superkingdoms (realms), 65 orders, 233 families, 2,606 genera, and more than 10,000 viral variants (strains) [5].

According to the well-known classification by D. Baltimore [6], which is based on the characteristics of the genomic nucleic acid (NA) and the strategy for its expression in an infected cell, viruses are divided into seven genetic classes: I. Double-stranded DNA viruses; II. Single-stranded (+)-sense DNA viruses; III. Double-stranded RNA viruses; IV. Singlestranded (+)-sense RNA viruses; V. Single-stranded (–)-sense RNA viruses; VI. Single-stranded (+)-sense RNA viruses with a DNA intermediate in their life cycle; and VII. Double-stranded DNA viruses with an RNA intermediate. This classification is based on the concept of positive-sense viral mRNAs; i.e., RNA molecules translated by cellular ribosomes to form viral proteins [7, 8]. Contrarywise, negative-sense RNAs encode and translate proteins through the intermediate synthesis of a complementary (positive-sense) mRNA strand. In genomic viral DNAs, a strand identical to the translated (+)-mRNA molecule is designated as a positive-sense strand, whereas a strand complementary to mRNA is designated as a negative-sense strand.

Differences in the viral genome structure and variations in the patterns of its expression in an infected cell (i.e., strategies for viral genome expression) underlie virus diversity, pantropic adaptation of viruses to various organisms such as bacteria, fungi, plants, fish, and animals, in particular humans, and ensure the global spread of viruses on Earth, and possibly in space and other planets [6].

The genetic diversity of viruses, which underlies the Baltimore classification, was considered as follows: one unique viral genome develops one genome strategy; i.e., one genome has one replication scheme and directs the formation of one structural and functional class of virions (i.e., one type of virus reproduction). This implies a uniform and unified process for the synthesis of viral particles (virions) within one viral genus (or family) [7, 8]. However, our discovery of unique genes in the genome of RNA viruses which are arranged according to the stacking principle (the so-called gene stacking) and encode proteins in opposite (ambipolar) directions, indicates the possibility of several alternative strategies for genome implementation in one virus, which leads to different structural classes of viral particles.

**Fig. 1:**
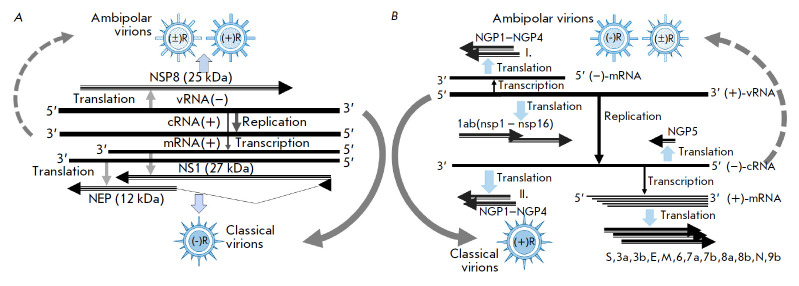
Localization of ambipolar genes in the RNA genome of the influenza A virus and coronavirus and the formation of ambipolar virions. (*A*) Scheme of gene coding in the influenza virus genome segment *NS *in the A/Aichi/2/68 (H3N2) model. The influenza virus has a negative-sense genome that encodes three proteins: negative-sense NS1 and NEP and the positive-sense stacking protein NSP8. The canonical pathway strategy for segment 8 (*NS*) is shown. This pathway is realized through synthesis of the NS1 and NEP proteins, formation of classical enveloped virions containing the PB1, PB2, PA, HA, NP, NA, M1, and M2 proteins, and a possible alternative pathway with the formation of the non-canonical (ambipolar) NSP8 protein and similar ambipolar proteins of positive-sense genes, found in the PB1, PB2, PA, NP, M, and NS segments (NSP1–NSP8 proteins, respectively, according to the numbering of RNA segments in the viral genome). Non-canonical ambipolar virions decorated with NSP1–NSP8 proteins have not yet been found and remain hypothetical in nature (dotted arrow). (*B*) Scheme of gene coding in the RNA genome of coronavirus in the SARS-CoV2 model. Coronavirus has a positive-sense genome encoding five major structural (S1/S2, N, E, M) and 16 (nsp 1–16) accessory non-structural polypeptides. The classical pathway of positive-sense strategy leads to the formation of classical enveloped virions containing the S1/S2, N, E, and M proteins (solid arrow). The negative genome direction (3’ → 5’) encodes extended open reading frames in complimentary positive polarity (5’ → 3’) RNA molecules possessing all essential elements, such as the initiator AUG, Kozak element, IRES, and stop codons. These translational frames (genes) are designated as negative gene proteins (NGPs), and the most extended NGPs, NGP1–GP5, have a molecular weight in the range of 7–0 kDa [17]. The dash arrow shows an alternative pathway of genome strategy with the formation of non-canonical (ambipolar) virions. The double arrow shows proteins and the direction of their coding in the genome. Ambipolar NGP1–GP5 polypeptides are synthetized through the formation of a subgenomic (–-mRNA and its translation (pathway I), and also through translation of a full-length complementary genomic (–-cRNA (pathway II)

In 2007, we analyzed the negative-sense genome of influenza A viruses (orthomyxovirus family) and found extended open reading frames (ORFs) that, unlike the canonical influenza virus genes (*PB1*, *PB2*, *PA*,* HA*, *NP*, *NA*, *M*, *NS*) with negative coding polarity in the genomic RNA in the 3’ → 5’ direction, had additional positive coding polarity (in the 5’ → 3’ direction of the genomic molecule) (*Fig. 1A*). The peculiarity of these ambipolar genes was their localization in genome regions overlapping the corresponding classical negative-sense genes; the so-called stacking arrangement [9, 10, 11, 12, 13, 14]. Later, in 2019, we identified extended open reading frames with a negative encoding direction (3’ → 5’) in the positive-sense RNA genome of coronaviruses [15, 16, 17, 18] (*Fig. 1B*). The ambipolar genes identified in the genomes of orthomyxo- and coronaviruses were found to be characterized by the presence of all the functional elements necessary for expression of these genetic frameworks as translational genes [19, 20]: ATG start codons (or an alternative CUG codon), translational stop codons [21], canonical initiation Kozak sequences in the initiation codon site (Kozak element [22]), and the presence of internal ribosome entry sites (IRESs) [23] possessing a typical secondary structure in the ambipolar gene start site. Computer analysis of algorithms of the viral genome primary structure revealed various structural and functional domains in the predicted protein products of ambipolar genes, in particular transmembrane elements of ion channel proteins, structural domains of ubiquitin dehydrogenase, and several domains typical of the proteins involved in immunity and inflammation regulation [9, 14, 18].

Today, the genome of one virus species (genus) is believed to have one strategy that determines the formation of viral particles of a certain (canonical) structure and a characteristic range of hosts. The discovery of ambipolar stacking genes in the genomes of RNA viruses suggests the existence of alternative strategies in the genome of one virus species (genus) whose expression pathways may (1) provide the synthesis of several structural and functional classes of virions that differ in both their protein composition and the structural form (polarity) of genomic RNA and/or (2) develop several different strategies for virus replication and its pathogenesis in an infected macroorganism. The presence of several strategies in one viral genome provides a reserve of viral adaptive properties, which may be considered as a pathway (or modification) of genetic bet-hedging (i.e., genetic rescue of viruses).

**Fig. 2:**
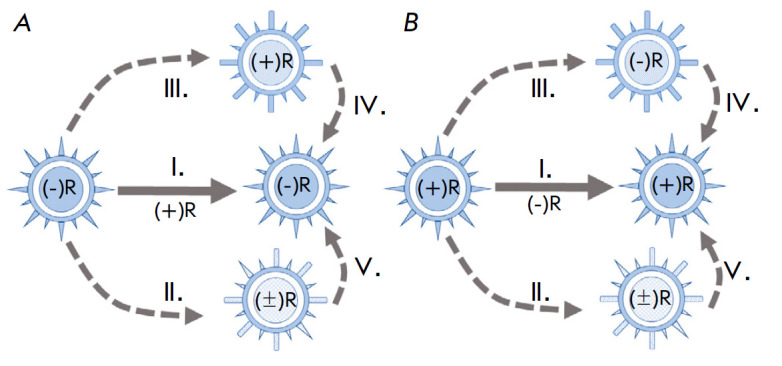
Alternative strategies of the influenza virus negative- sense genome and the formation of ambipolar virions. The diagram illustrates the alternative strategies of the viral genome using the influenza virus (*A*) and coronavirus (*B*) genome models and is applicable to other viruses (pneumo-, paramyxo-, rhabdo-, filoviruses, etc.) possessing a negative-sense RNA genome (–R). Genome strategy is outlined as a viral genome replication pathway leading to the formation of canonical viral particles of a given structure and composition, both in terms of viral genome polarity and protein composition of the viral envelope. Three alternative strategies possible for one unique viral genome are shown. Currently, pathway 1 is considered as canonical, while four other strategies remain hypothetical. Probably, in a given biochemical context of infected cells, strategies II–V may be implemented, when full-length genomic RNA chains ((+)R and (±)R) are packaged by proteins of distinct compositions (denoted by different symbols ( , , , ), including proteins of ambipolar genes. In this case, different virion types may have different envelope structures with/without cellular lipids, the so-called enveloped and non-enveloped virions. Genetic realization of viral genome replication is performed by RNA-dependent polymerase that can be included in the virion and provide the beginning of viral replication in the target cell. (+) R, (–)R, and (±)R are three possible variants of a progeny virion genomic RNA with a single-stranded positive/negative sense and double-stranded structure, respectively. Possible pathways to alter the genome expression strategy in one species of virus are shown by dotted arrows and labels (II–V); the classical pathway of the negative-sense strategy for the influenza virus is shown by the main arrow (I), respectively. A targeted search for the virions of the indicated non-canonical structural classes II–V is required to pinpoint strategies II–V

The multiple strategies of the genome in one virus species (genus) and the expression schemes of its classical and alternative strategies are shown in *Fig. 2* for the influenza virus and coronavirus models. The influenza virus comprising genomic (–)-RNA is characterized by the possibility of both a classical pathway of genome implementation (pathway I; central arrow in *Fig. 2A*) and alternative strategies (*Fig. 2*, II–V). Implementation of alternative genome strategies may lead to the formation of ambipolar virions that may contain both classical proteins (PB1, PA, PB2, HA, NA, NP, M1, M2) and additional proteins–products of the ambipolar genes *NSP1*–*NSP8 *(*NSP *– Negative Strand Protein) of appropriate genomic RNA segments (*Fig. 2A*). Expression of the classic coronavirus strategy also leads to the formation of virions containing the canonical (+)-RNA genome and classical structural proteins: N (nucleocapsid protein), S (surface glycoprotein), E (membrane protein), and M (internal matrix protein) and a number of auxiliary non-structural regulatory proteins (nsp1-nsp16) that support viral replication in target cells and suppression of the host’s immune response. However, the products of the main ambipolar genes *NGP1*–*NGP5 *(negative gene proteins [17]), which may form a new structural class of virions (the so-called ambipolar virions; *Fig. 2B*, dotted arrow), escape the attention of researchers. So far, these proteins encoded by open ambipolar genes have not been found in infected cells. A possible reason lies in either the minor level of their synthesis or their strictly selective expression only in specialized body cells containing the unique factors necessary for the expression of these viral stacking genes under certain conditions of the intracellular and/or surrounding extracellular environment. At the same time, there are indirect approaches to observe ambipolar gene expression in an infected macroorganism. Animals infected with the influenza A virus were found to develop clones of cytotoxic lymphocytes that recognize specific peptide domains of the influenza virus ambipolar proteins, in particular the NSP8 protein encoded by the ambipolar *NSP8 *gene of the influenza A virus* NS *segment [24, 25, 26].

We may posit that these non-canonical proteins are able to decorate the viral genome from a new class of viral particles performing unique regulatory functions, and altering the virus behavior in an infected organism; e.g., switching from productive virus infection to a latent persistent (low reproductive) viral infection process. Furthermore, there may be an alternative when a genome molecule becomes an RNA chain complementary (ambipolar replica) to the canonical virus genome: the coronavirus (–)RNA or influenza virus (+)RNA (*Fig. 2*). Thus, ambipolar viral particles may contain both ambipolar proteins and ambipolar genomic RNA replicas, providing an alternative pathway for the viral genome strategy. As a result, one unique viral genome may be implemented in several, alternative strategies – with or without involvement of ambipolar genes – and viruses may possess several possible life pathways, depending on the context of the surrounding cellular processes. This idea is illustrated in *Fig. 2*. This multivariant mechanism of a unique viral genome strategy may be considered as a way of bet-hedging by viruses, which promotes the establishment of alternative ways of virus replication and the creation of reserve adaptive potentials for viruses of various families. In this aspect, RNA viruses may be similar to DNA viruses and RNA-containing retroviral (virus-like) transposons that have a dual-track lifestyle: as a DNA provirus and a mature virus, respectively, which determines the vertical (a viral genome DNA copy integrated into the cell genome) and horizontal (mature virions) ways of their existence in the host, depending on the propagation environment and the range of hosts [27, 28, 29].

The ambipolar genes of viruses are endowed with high evolution stability. In particular, in the natural population of highly variable influenza viruses, these genes have been observed in the genome with all the necessary regulatory elements for more than 100 years, despite a noticeable population variability in both canonical and identified ambipolar genes with a characteristic high dN/dS coefficient that indicates pronounced immunological pressure from the host macroorganism in nature [14]. The evolutionary stability of ambipolar genes in the natural population of viruses emphasizes the vital role of these genes for the virus and, therefore, resistance to natural restrictive selection. The presence of ambipolar genes in the genome of RNA-containing viruses provides a new pathway for the formation of viral diversity, when virions possessing an identical genome may vary in the expression scheme (strategy) of the genome and have different replication pathways that provide variations both in the composition of the proteins expressed by “positive” or “negative” genes (the so-called ambipolar virions) and in genome polarity [17]. Alternative genome strategies and a change in the profile of synthesized proteins and the viral envelope give the virus additional opportunities to adapt to a new host and extend a host’s range of viruses. In this case, a virus can not only use different strategies to express its genome, but also change these strategies depending on the host, which is illustrated in *Fig. 2* (dotted arrows). So far, these pathways of multiple expression strategy of the viral genome remain as hypothetical and enigmatic as the “dark side of the Moon.” Experimental verification of this crystal-ball reading exercise will enable us to evaluate the possible existence of ambipolar classes of stealth virions hidden from the eye of researchers. To date, mature protein products encoded by identified ambipolar viral genes in an infected organism have not yet been detected. But this does not mean that expression of these viral stacking genes is not implemented in nature. Identification of the expression of these genes requires a targeted search using original approaches and highly sensitive methods for identifying proteins in various organs and the specific cells of an infected host macroorganism. It is possible that the unraveling of alternative strategies of viral genomes may be important for understanding virus evolution and the pathogenesis of viral infections, as was the case in covid-2019 when long-term and severe complications of the viral infection could develop due to the formation of ambipolar virions hidden from the attention of researchers and medical practitioners.

**Fig. 3:**
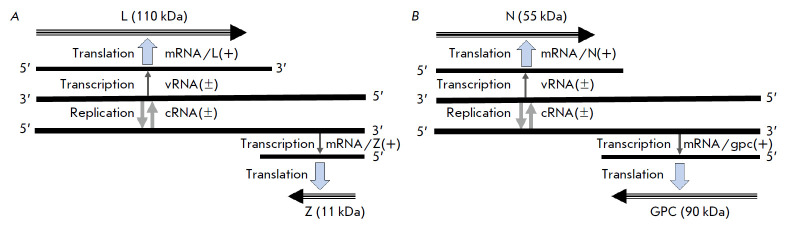
Schematic diagram of the bipolar (ambisense) strategy of the arenavirus genome (Arenaviridae family; Mammarenavirus genus). The arenavirus genome (lymphocytic choriomeningitis virus (LCMV); ac.n. AY847350; AY847351) is used. The family combines pathogens of severe human hemorrhagic fevers (Lassa, Lujo, Machupo, Junin, Chapare, Guanarito, Sabia, etc.). The arenavirus genome contains four genes that encode: (*A*) polymerase protein (L, 110 kDa) and non-structural multifunctional protein (Z, 11 kDa); (*B*) nucleocapsid protein (N, 55 kDa) and surface glycoprotein (GPC; 90 kDa) [31]. Coding of the *L *and *N *genes has negative polarity, and that of the *GPC *and *Z *genes has opposite (positive) polarity. All four genes are uncoupled in the arenavirus genome and do not overlap, and expression of each of the genes in infected cells requires the synthesis of individual 5’-capped mRNAs

Obviously, the ambipolar stacking of genes found in RNA viruses provides the virus with, first, an enhanced information capacity of the genome. Second, it underlies the linked (reciprocal) evolution of viral genes when mutations in one gene generate changes in a stacking gene and, thus, represent a kind of genetic synteny. Third, the protein products of stacked genes may be functionally linked and have a predetermined structural correspondence to each other, which remains a hypothetical and requires experimental evidence [14, 17]. The gene-stacking trait distinguishes these viruses from the known four genera of ambipolar viruses (tospo-, phlebo-, arena-, and bunyaviruses), in which ambipolar genes are located separately in the genome, without overlapping with other genes, and function as the main genes that drive the synthesis of the major structural and regulatory viral proteins [30]. This strategy of the viral genome with separated ambisense genes devoid of stacking localization is shown in *Fig. 3* using an arenavirus model (Arenaviridae family, Mammalovirus genus). In this regard, the difference in stacking allows us to consider two major groups of ambipolar viruses. To date, the following division seems logical: in the first group of viruses (influenza viruses, coronaviruses) with gene stacking in the viral genome, pathways of ambipolar genome strategies may have an alternative (optional) character, while in viruses lacking gene stacking (tospo-, phlebo-, arena-, and bunyaviruses), the implementation of the ambisense genome strategy should be considered as an obligatory (mandatory) reality for virus replication. Further targeted search for the expression pathways of alternative genome strategies in one viral species and identification of a hypothetical class of ambipolar virions will answer the question of the existence of this type of viral life diversity and its role in the evolution of viruses of various genera. This knowledge will come handy in the development of new vaccines and antiviral drugs and add to our understanding of the molecular basis of viral disease pathogenesis.
